# Prognostic value of combined coronary CT angiography and myocardial perfusion imaging in women and men

**DOI:** 10.1093/ehjci/jead072

**Published:** 2023-04-22

**Authors:** Iida Kujala, Wail Nammas, Teemu Maaniitty, Iida Stenström, Riku Klén, Jeroen J Bax, Juhani Knuuti, Antti Saraste

**Affiliations:** Turku PET Centre, Turku University Hospital and University of Turku, Kiinamyllynkatu 4-8, FI-20520 Turku, Finland; Turku PET Centre, Turku University Hospital and University of Turku, Kiinamyllynkatu 4-8, FI-20520 Turku, Finland; Heart Center, Turku University Hospital and University of Turku, Hämeentie 11, FI-20520 Turku, Finland; Turku PET Centre, Turku University Hospital and University of Turku, Kiinamyllynkatu 4-8, FI-20520 Turku, Finland; Department of clinical physiology, nuclear medicine and PET, Turku University Hospital, Turku, Finland; Turku PET Centre, Turku University Hospital and University of Turku, Kiinamyllynkatu 4-8, FI-20520 Turku, Finland; Heart Center, Turku University Hospital and University of Turku, Hämeentie 11, FI-20520 Turku, Finland; Turku PET Centre, Turku University Hospital and University of Turku, Kiinamyllynkatu 4-8, FI-20520 Turku, Finland; Heart Center, Turku University Hospital and University of Turku, Hämeentie 11, FI-20520 Turku, Finland; Department of Cardiology, Leiden University Medical Center, Leiden, The Netherlands; Turku PET Centre, Turku University Hospital and University of Turku, Kiinamyllynkatu 4-8, FI-20520 Turku, Finland; Department of clinical physiology, nuclear medicine and PET, Turku University Hospital, Turku, Finland; Turku PET Centre, Turku University Hospital and University of Turku, Kiinamyllynkatu 4-8, FI-20520 Turku, Finland; Heart Center, Turku University Hospital and University of Turku, Hämeentie 11, FI-20520 Turku, Finland

**Keywords:** chronic chest pain, computed tomography angiography, coronary artery disease, positron emission tomography, hybrid imaging, sex

## Abstract

**Aims:**

Combined anatomical and functional imaging enables detection of non-obstructive and obstructive coronary artery disease (CAD) as well as myocardial ischaemia. We evaluated sex differences in disease profile and outcomes after combined computed tomography angiography (CTA) and positron emission tomography (PET) perfusion imaging in patients with suspected obstructive CAD.

**Methods and results:**

We retrospectively evaluated 1948 patients (59% women) referred for coronary CTA due to suspected CAD during the years 2008–2016. Patients with a suspected obstructive lesion on coronary CTA (*n* = 657) underwent ^15^O-water PET to assess stress myocardial blood flow (MBF). During a mean follow-up of 6.8 years, 182 adverse events (all-cause death, myocardial infarction, or unstable angina) occurred. Women had more often normal coronary arteries (42% vs. 22%, *P* < 0.001) and less often abnormal stress MBF (9% vs. 28%, *P* < 0.001) than men. The annual adverse event rate was lower in women vs. men (1.2% vs. 1.7%, *P* = 0.02). Both in women and men, coronary calcification, non-obstructive CAD, and abnormal stress MBF were independent predictors of events. Abnormal stress MBF was associated with 5.0- and 5.6-fold adverse event rates in women and men, respectively. There was no interaction between sex and coronary calcification, non-obstructive CAD, or abnormal stress MBF in terms of predicting adverse events.

**Conclusion:**

Among patients evaluated for chronic chest pain, women have a lower prevalence of ischaemic CAD and a lower rate of adverse events. Combined coronary CTA and PET myocardial perfusion imaging predict outcomes equally in women and men.

## Graphical Abstract

**Graphical Abstract:**
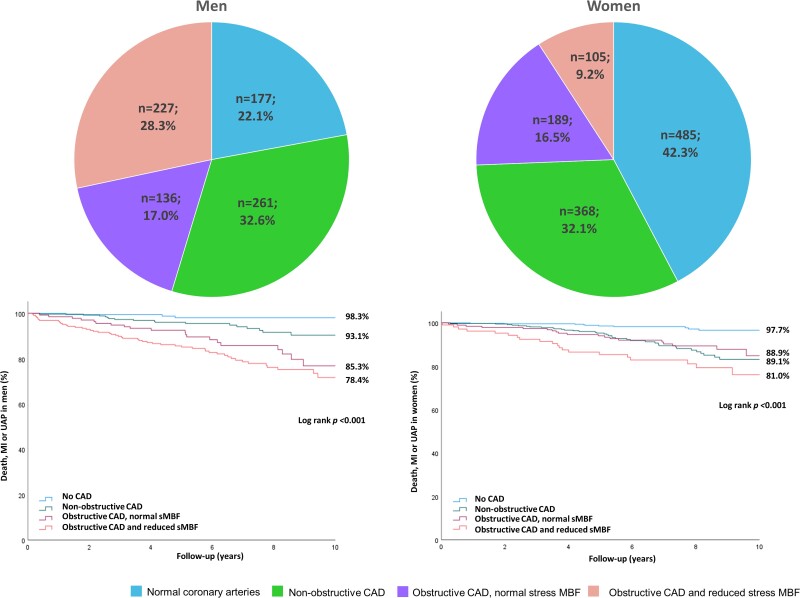

## Introduction

There are differences in the diagnostic accuracy of non-invasive tests to assess obstructive coronary artery disease (CAD) between women and men.^1–3^ Recent comparative studies indicate that in women anatomic evaluation by coronary computed tomography angiography (CTA) is associated with a lower rate of CAD diagnosis than noninvasive functional tests as compared to men.^4–6^ Furthermore, women may gain similar or even more prognostic information from coronary CTA than stress testing for ischaemia, whereas men benefit equally from both of these testing modalities.^4–6^

Hybrid or combined imaging using coronary CTA and functional testing for ischaemia enables accurate detection of non-obstructive and obstructive CAD as well as assessment of the haemodynamic significance of coronary stenosis.^7,8^ Clinical practice guidelines recommend functional evaluation of CAD before revascularization decisions.^9^ We have constructed an observational registry of patients in whom the haemodynamic significance of any suspected obstructive stenosis detected by coronary CTA was routinely evaluated by ^15^O-water positron emission tomography (PET) myocardial perfusion imaging.^10^ We previously demonstrated that normal stress myocardial blood flow (sMBF) despite obstructive CAD on CTA, is associated with favourable prognosis.^10^ However, the prognostic value of combined anatomical and functional imaging in men vs. women remains unknown.

We hypothesized that a detailed assessment of CAD by combining anatomical and functional imaging reveals differences in characteristics of CAD, while the prognostic value of diagnostic testing in women vs. men with chronic chest pain would be the same. For this purpose, we compared the findings and prognostic value of combined coronary CTA and ^15^O-water PET perfusion imaging among women and men with suspected CAD.

## Methods

### Study cohort

We retrospectively identified all consecutive patients referred for coronary CTA in the Turku PET Centre due to suspected obstructive CAD in the period of 2008–2016. We did not include patients with known CAD (previous coronary revascularization or ≥50% diameter stenosis on invasive coronary angiography, ICA) or those referred primarily for reasons other than suspected symptomatic CAD, such as aetiological evaluation of heart failure or pre-operative evaluation. In cases of repeat tests during the study period, only the earliest test was included in the current analysis.

**Figure 1:**
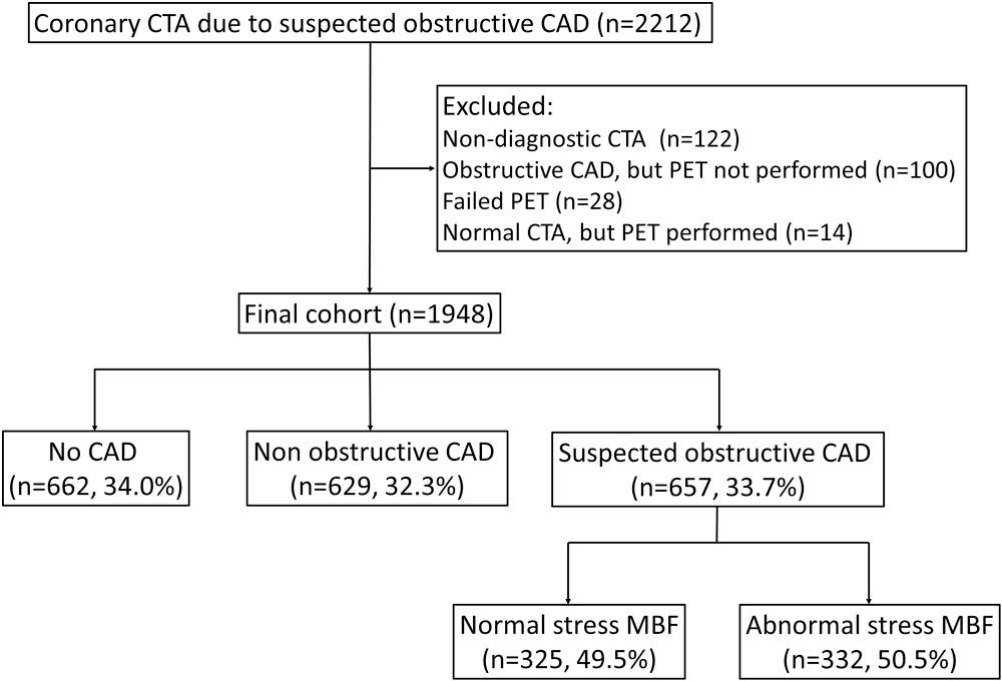
Flow chart of patient selection and classification according to imaging findings.

As previously described,^10,11^ it is routine practice in our hospital that patients initially undergo coronary CTA using a hybrid PET-CT scanner, and immediately after coronary CTA, the attending physician evaluates the CTA scan to decide whether to perform PET myocardial perfusion imaging during the same visit. If the coronary CTA reveals obstructive CAD (diameter stenosis ≥50%), the haemodynamic significance of lesions is evaluated using ^15^O-water PET during adenosine stress. Out of the 2212 patients identified, we excluded 122 patients who did not complete the combined imaging protocol for reasons outlined in *Figure 1*, including 61 patients referred directly for ICA without PET. Thus, the final analysis included 1948 patients.

The study complies with the Declaration of Helsinki. The Ethics Committee of the Hospital District of Southwest Finland approved the study protocol and waived the need for written informed consent by patients. The Finnish Institute for Health and Welfare gave permission to the retrospective collection of clinical data.

### Coronary CTA and PET image acquisition and interpretation

Coronary CTA and PET scans were performed using a 64-row hybrid PET-CT scanner (GE Discovery VCT or GE D690, General Electric Medical Systems, Waukesha, Wisconsin) using previously described procedures.^7,10^

Coronary artery calcium (CAC) scans were performed before coronary CTA in the majority of patients. Based on the CAC score, patients were categorized into those with no coronary calcification (CAC score = 0), mild calcification (CAC score = 1–99), moderate calcification (CAC score = 100–399), and extensive calcification (CAC score ≥400).

Before coronary CTA, metoprolol was given intravenously to achieve a target heart rate of <60 beats/min. Isosorbide dinitrate aerosol (1.25 mg) was administered for coronary artery vasodilation. Coronary CTA was performed using an intravenously administered low-osmolal iodine contrast agent followed by a saline flush. The prospectively triggered acquisition was applied whenever feasible. Collimation was set at 64 × 0.625 mm, gantry rotation time was 350 ms, tube current 600–750 mA, and voltage 100–120 kV, depending on patient size. Coronary CTA scans were analysed in a standardized fashion, reporting the presence of atherosclerotic plaque and obstructive stenosis (≥50% in diameter) for each coronary artery segment.

Before the PET scan, patients fasted overnight and abstained from alcohol and caffeine for 24 h. Adenosine infusion started 2 min before the stress PET scan and continued at a rate of 140 μg/kg/min until the scan was complete. ^15^O-water (Radiowater Generator, Hidex Oy, Turku, Finland) was injected as an intravenous bolus (mean injected activity 900–1100 MBq) over 15 s followed by dynamic PET acquisition (14 × 5 s, 3 × 10 s, 3 × 20 s, and 4 × 30 s).

The PET data were analysed quantitatively using Carimas software (developed at Turku PET Centre, Turku, Finland). Absolute sMBF (mL/g/min) was quantified individually for each of the standard 17 myocardial segments. Basal septal segments (segments 2 and 3) were excluded from the analysis. Abnormal stress MBF was defined as ≤2.3 mL/g/min in ≥1 segment based on previous research.^12^

Based on both CTA and PET findings we classified patients into four categories: (i) normal coronary arteries; (ii) non-obstructive CAD based on CTA; (iii) obstructive CAD by CTA and normal sMBF; and (iv) obstructive CAD by CTA and abnormal sMBF.

### Clinical and follow-up data collection

Data on traditional cardiovascular risk factors (smoking, diabetes mellitus, hypertension, dyslipidemia, and family history of CAD), symptoms, and medications were retrospectively collected from electronic medical records.

The primary outcome was a composite of all-cause death, non-fatal myocardial infarction (MI), or unstable angina pectoris (UAP) based on comprehensive data until May 2020 in the registries of the Finnish Institute for Health and Welfare and the Centre for Clinical Informatics of the Turku University Hospital. Investigators used electronic medical records to validate all adverse events. Data of early revascularization within 6 months after coronary CTA, with either percutaneous coronary intervention (PCI) or coronary artery bypass graft (CABG) surgery, were also collected, but not used as endpoints. In case of the occurrence of multiple adverse events, the first event was considered.

### Statistical analysis

Continuous variables are reported as mean and standard deviation (SD) or median [interquartile range], as appropriate. Categorical variables are shown as count (percentage). The χ^2^ test, Fisher Exact test, student *t*-test, and Mann–Whitney U test were used as appropriate. The cumulative incidence of events was based on Kaplan–Meier estimates and was compared between women and men using the log-rank test. Cox proportional hazards models were used to identify the univariable and multivariable predictors in the whole cohort, men, and women. The clinical variables bearing significant association with the composite outcome in the univariable analysis were added to the clinical multivariable model as covariates. Those covariates bearing significant association with the composite outcome in the clinical multivariable model were then transferred as covariates to multivariable models including CAC or PET-CTA findings. The prognostic value of the multivariable models to predict the composite outcome was tested using the receiver operating characteristics (ROC) curve analysis and time-dependent ROC as implemented in the R package time ROC.^13^ The statistical difference between the ROC curves was determined using the DeLong method^14^ using R package pROC.^15^ Furthermore, the interaction between sex on one hand and either the CAC or PET-CTA findings, on the other hand, was tested using Cox proportional hazards models. Statistical significance was set at *P* < 0.05. Statistical analyses were performed using SPSS v. 25.0 (IBM Corporation, New York, USA) statistical software and R statistical computing environment version 3.6.0 (R Foundation for Statistical Computing, Vienna, Austria. URL https://www.R-project.org/).

## Results

The cohort consisted of 1948 patients including 1147 (58.9%) women with suspected obstructive CAD who underwent coronary CTA. In 1291 (66.3%) patients, obstructive CAD was excluded based on coronary CTA alone, whereas 657 (33.7%) had ^15^O-water PET myocardial perfusion imaging to evaluate haemodynamic significance of suspected obstructive coronary stenosis. As shown in *Figure 1*, 34.0% of patients had normal coronary arteries, 32.3% had non-obstructive CAD, 16.7% had obstructive CAD on coronary CTA and normal sMBF, and 17.0% had obstructive CAD and abnormal sMBF. Supplementary data online, *Figure S1* shows an example of findings.

### Baseline characteristics of men vs. women

Characteristics of patients are shown in *Table 1*. Women were slightly older, and had less frequently diabetes, and more frequent family history of premature CAD than men. Women were less likely to have a smoking history than men, but there was no difference in body mass index or the prevalence of either dyslipidemia or hypertension. There were only four women and eight men with known left ventricular systolic dysfunction (ejection fraction <40%).

**Table 1:** Clinical characteristics, imaging findings, and early invasive therapies of all patients, men, and women

	Cohort (*n* = 1948)	Men (*n* = 801)	Women (*n* = 1147)	*P* value
Age (years)^a^	62.9 ± 9.6	59.9 ± 10.6	63.4 ± 9.3	<0.001
Body mass index (kg/m^2^)^a^	27.9 ± 6.6	27.9 ± 5.7	28.0 ± 7.3	0.6
Current or ex-smoking	625 (32.1)	338 (42.2)	287 (25.0)	<0.001
Diabetes mellitus	288 (14.8)	137 (17.1)	151 (13.2)	0.01
Hypertension	1083 (55.6)	455 (56.8)	628 (54.8)	0.3
Dyslipidemia	1221 (62.7)	497 (62.0)	724 (63.1)	0.6
Family history of CAD	918 (47.1)	301 (37.6)	617 (53.8)	<0.001
Typical chest pain	411 (22.1)	153 (20.0)	258 (23.6)	
Atypical/non-cardiac chest pain	963 (49.4)	371 (46.3)	592 (51.6)	
Dyspnoea	755 (38.8)	261 (32.6)	494 (43.1)	<0.001
Coronary artery calcium score (*n* = 1594)				<0.001
0	578 (36.3)	152 (23.2)	426 (45.3)	
1–99	467 (29.3)	185 (28.3)	282 (30.0)	
100–399	304 (19.1)	159 (24.3)	145 (15.4)	
> 400	245 (15.4)	158 (24.2)	87 (9.3)	
Coronary CTA and PET findings				<0.001
No CAD	662 (34.0)	177 (22.1)	485 (42.3)	
Non-obstructive CAD	629 (32.3)	261 (32.6)	368 (32.1)	
Obstructive CAD and normal sMBF	325 (16.7)	136 (17.0)	189 (16.5)	
Obstructive CAD and abnormal sMBF	332 (17.0)	227 (28.3)	105 (9.2)	
Invasive coronary angiography	217 (11.1%)	140 (17.5)	77 (6.7)	<0.001
Early revascularization (6 months)	127 (6.5%)	86 (10.7%)	41 (3.6%)	<0.001
PCI	112 (5.7%)	74 (9.2)	38 (3.3)	<0.001
CABG	18 (0.9%)	15 (1.9)	3 (0.3)	<0.001
Follow-up events				
Death	126 (6.5)	60 (7.5)	66 (5.8)	0.1
MI	45 (2.3)	23 (2.9)	22 (1.9)	0.1
UAP	21 (1.1)	11 (1.4)	10 (0.9)	0.2
Death or MI	164 (8.4)	80 (10.0)	84 (7.3)	0.03
Death, MI or UAP	182 (9.4)	90 (11.3)	92 (8.0)	0.01

mean ± standard deviation (range).

CAD, coronary artery disease; CABG, coronary artery bypass grafting; CTA, computed tomography angiography; MI, myocardial infarction; PCI, percutaneous coronary intervention; PET, positron emission tomography; sMBF, stress myocardial blood flow; UAP, unstable angina pectoris.

The most common presenting symptom was atypical angina or non-anginal chest pain. Typical angina was more common in women than men. Women also reported dyspnoea more often than men.

### Sex differences in coronary CTA and PET perfusion findings

**Figure 2:**
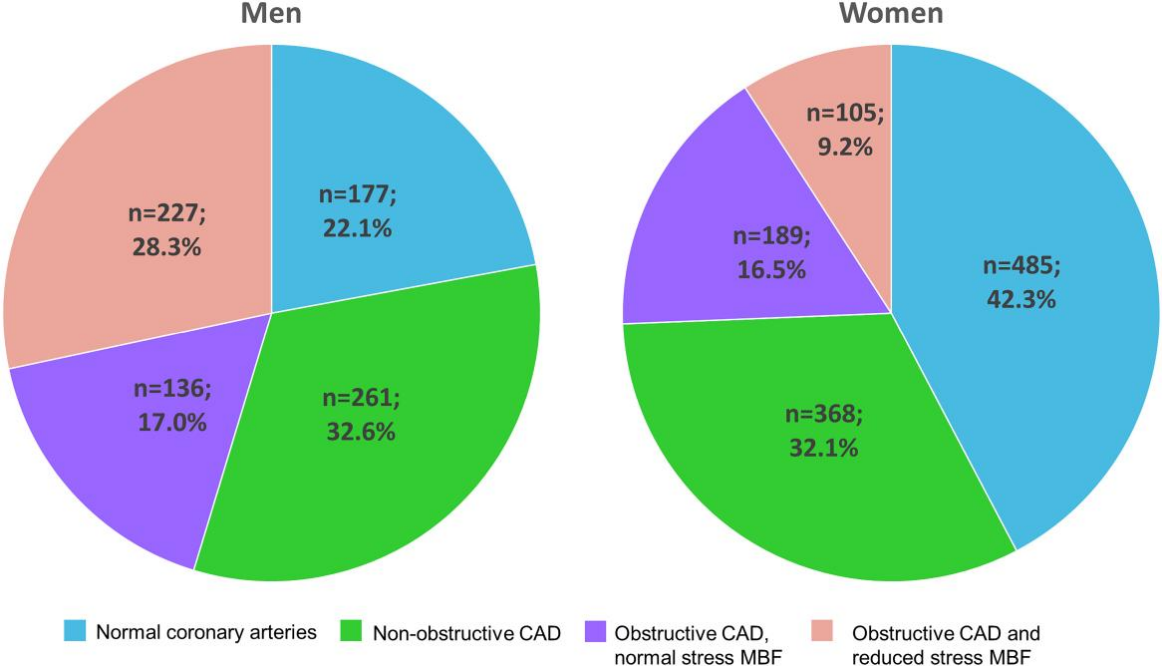
Graphs showing proportions of women and men with normal coronary arteries, non-obstructive CAD, obstructive CAD and normal sMBF, and obstructive CAD and abnormal sMBF.

Women were more likely to have normal coronary arteries than men (42.3% vs. 22.1% *P* < 0.001) while abnormal sMBF was less frequent in women than men (9.2% vs. 28.3% *P* < 0.001, *Table 1* and *Figure 2*). Hemodynamically non-significant CAD was present similarly in women and men (48.6% vs. 49.6%). However, the CAC score was higher in men than women.

### Follow-up

The mean time of follow-up was 6.8 ± 2.5 years with a median of 6.65 years. During the follow-up, there were 126 deaths, 45 MIs, and 21 UAPs. The composite outcome of death, MI, or UAP occurred in 182 patients and was less frequent in women than men (8.0% vs. 11.3%, *P* = 0.01, *Table 1*).

Most (190 out of 217) early ICAs were performed in patients with ischaemic CAD (obstructive CAD on CTA and abnormal sMBF). Similarly, most revascularizations were performed in patients with ischaemic CAD (123 out of 127). Among 332 patients with ischaemic CAD, there was no difference in the ICA referral rate between women and men (60.0% vs. 55.9%, *P* = 0.4). A similar proportion of women and men with ischaemic CAD had early revascularization (36.2 vs. 37.4%, *P* = 0.8), and the revascularization rate among those undergoing ICA was similar (53.2% vs. 61.4%, *P* = 0.2). Among patients with ischaemic CAD, there was no significant difference in the cumulative incidence of events at long-term follow-up between patients who underwent early revascularization (17.9%) vs. were treated conservatively (22.5%, *P* = 0.4).

### Predictors of events in women and men

Male sex was an independent risk factor for adverse events. Age and dyspnoea were associated with adverse events in women, whereas in men, age, diabetes, hypertension, and typical angina predicted events (*Table 3*).

Coronary calcification was an independent predictor of events after adjustment for age, clinical risk factors, and symptoms both in women and men (*Tables 2* and *3*). Extensive coronary calcification (CAC score ≥400) predicted events with an adjusted hazard ratio of 20.07 (95%CI 2.63–153.10) in men and 4.94 (95%CI 2.31–10.58) in women, when compared to patients without coronary calcification (CAC score 0).

**Table 2:** Univariable predictors of adverse events (all-cause death, myocardial infarction, or unstable angina pectoris) in all patients, men and women

	All patients		Men		Women	
	HR (95% CI)	*P* value	HR (95% CI)	*P* value	HR (95% CI)	*P* value
Age	1.07 (1.05–1.09)	<0.001	1.07 (1.04–1.09)	<0.001	1.08 (1.05–1.11)	<0.001
Male sex	1.39 (1.04–1.86)	0.02				
DM	1.68 (1.18–2.40)	0.004	2.17 (1.37–3.43)	<0.001	1.13 (0.63–2.04)	0.6
Hypertension	1.86 (1.36–2.56)	<0.001	2.11 (1.33–3.35)	0.001	1.64 (1.07–2.54)	0.02
Smoking	1.23 (0.91–1.66)	0.1	1.32 (0.87–1.99)	0.1	0.98 (0.61–1.57)	0.9
Dyslipidemia	0.97 (0.72–1.32)	0.8	1.07 (0.69–1.64)	0.7	0.90 (0.59–1.38)	0.6
Typical chest pain	1.52 (1.09–2.14)	0.01	2.00 (1.25–3.20)	0.004	1.21 (0.74–1.96)	0.4
Any chest pain	0.88 (0.63–1.24)	0.4	0.90 (0.56–1.42)	0.6	0.94 (0.56–1.56)	0.8
Dyspnoea	1.88 (1.41–2.52)	<0.001	1.69 (1.11–2.56)	0.01	2.31 (1.51–3.53)	<0.001
CAC						
0	Reference		Reference		Reference	
1–99	3.23 (1.74–6.01)	<0.001	11.52 (1.50–88.10)	0.01	2.68 (1.35–5.32)	<0.001
100–399	5.42 (2.92–10.06)	<0.001	23.24 (3.12–172.92)	0.002	3.73 (1.77–7.85)	<0.001
≥400	10.11 (5.61–18.23)	<0.001	40.48 (5.53–295.91)	<0.001	7.20 (3.55–14.59)	<0.001
PET-CTA						
No CAD	Reference		Reference		Reference	
Non-obstructive CAD	4.57 (2.55–8.19)	<0.001	4.21 (1.24–14.30)	0.02	5.17 (2.65–10.09)	<0.001
Obstructive CAD, normal sMBF	6.01 (3.28–11.03)	<0.001	9.30 (2.76–31.31)	<0.001	4.75 (2.29–9.87)	<0.001
Obstructive CAD, abnormal sMBF	10.01 (5.63–17.79)	<0.001	13.56 (4.22–43.52)	<0.001	8.04 (3.85–16.80)	<0.001

HR for age is per 1 year.

CAC, coronary artery calcium, CAD, coronary artery disease, CI, confidence interval, DM, diabetes mellitus, HR, hazard ratio.

**Table 3:** Multivariable predictors of adverse events (all-cause death, myocardial infarction, or unstable angina pectoris) in all patients, men and women

	All patients			Men			Women		
Model	HR (95% CI)	*P* value	AUC	HR (95% CI)	*P* value	AUC	HR (95% CI)	*P* value	AUC
***Clinical***			0.706			0.728			0.705
Age	1.06 (1.04–1.08)	<0.001		1.06 (1.03–1.08)	<0.001		1.07 (1.04–1.10)	<0.001	
Male sex	1.81 (1.32–2.46)	<0.001							
Diabetes				1.77 (1.09–2.89)	0.03				
Hypertension	1.61 (1.15–2.25)	0.004		2.03 (1.21–3.40)	0.009				
Typical angina	1.55 (1.10–2.18)	0.01		2.01 (1.24–3.25)	0.01				
Dyspnoea	1.60 (1.17–2.18)	0.003					1.88 (1.22–2.89)	0.004	
***Clinical + CAC***			0.730			0.746			0.720
Age	1.03 (1.01–1.05)	0.007		1.03 (1.00–1.06)	0.04		1.03 (1.00–1.07)	0.03	
Diabetes				1.76 (1.00–3.10)	0.04				
Dyspnoea	1.50 (1.05–2.04)	0.02					1.77 (1.08–2.89)	0.02	
CAC									
0	reference			reference			reference		
1–99	2.75 (1.44–5.27)	0.002		7.83 (1.00–61.12)	0.04		2.11 (1.04–4.27)	0.03	
100–399	4.23 (2.19–8.15)	<0.001		14.11 (1.84–108.01)	0.01		2.67 (1.22–5.80)	0.01	
≥ 400	7.20 (3.80–13.65)	<0.001		20.07 (2.63–153.10)	0.004		4.94 (2.31–10.58)	<0.001	
***Clinical + PET-CTA***			0.743			0.753			0.753
Age	1.05 (1.03–1.07)	<0.001		1.05 (1.02–1.08)	<0.001		1.05 (1.02–1.08)	<0.001	
Hypertension				1.81 (1.08–3.03)	0.02				
Dyspnoea	1.52 (1.11–2.08)	0.009					1.75 (1.13–2.69)	0.01	
PET-CT findings									
No CAD	Reference			Reference			Reference		
Non-obstructive CAD	3.36 (1.78–6.37)	<0.001		2.24 (0.64–7.80)	0.2		3.63 (1.83–7.20)	<0.001	
Obstructive CAD, normal sMBF	3.85 (1.97–7.52)	<0.001		4.37 (1.26–15.11)	0.02		2.99 (1.40–6.38)	0.005	
Obstructive CAD, abnormal sMBF	5.84 (3.04–11.21)	<0.001		5.61 (1.69–18.57)	0.005		4.99 (2.33–10.70)	<0.001	

The models are adjusted for significant clinical factors, CAC scores, and PET-CTA findings separately.

HR for age is per 1 year. *P*-values for comparing AUC values between models (clinical + CAC and clinical + PET-CTA) were 0.477 for all patients, 0.625 for men and 0.394 for women.

CAD, coronary artery disease; CAC, coronary calcium; CI, confidence interval; HR, hazard ratio; sMBF, stress myocardial blood flow.

Findings of PET-CTA were predictors of events after adjusting for age, clinical risk factors, and symptoms both in women and men (*Table 3*). As compared to patients with normal coronary arteries, the presence of abnormal sMBF predicted events with an adjusted hazard ratio of 5.61 (95%CI 1.69–18.57) in men and 4.99 (95%CI 2.33–10.70) in women. Non-obstructive CAD predicted adverse events in women, but the effect was statistically non-significant in men (*Table 3*).

**Figure 3:**
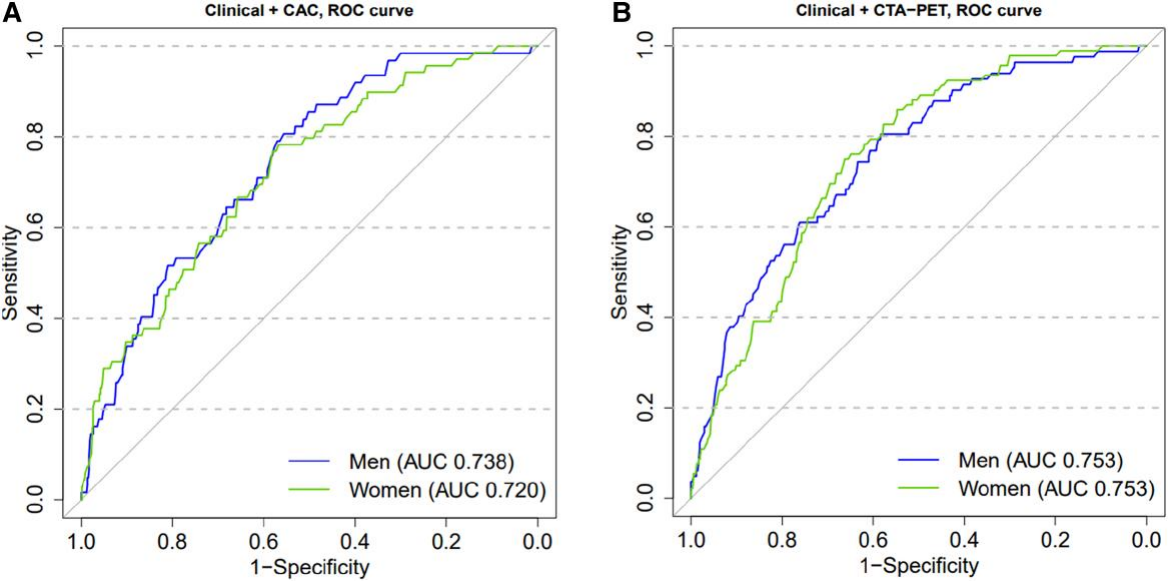
Receiver operating characteristic (ROC) curves showing no difference in the performance of coronary artery calcium (CAC) score (*P* = 0.687) (*A*) or combined coronary CTA and ^15^O-water PET (*P* = 0.999) (*B*) in predicting composite end-point of death, non-fatal MI or UAP between men and women. Analyses were adjusted for age, hypertension, and symptom status (typical angina and dyspnoea).

Based on Cox proportional hazards models, there was no significant interaction between sex and either coronary calcification or combined coronary CTA and PET findings (*P* = 0.4, and *P* = 0.2, respectively) in predicting adverse events. Similarly, based on the ROC analyses the prognostic value of adjusted coronary artery calcification or combined coronary CTA and PET to predict composite outcome was similar in males and females (*P* = 0.687 and *P* = 0.999, *Figure 3* and Supplementary data online, *Figure S2*). Overall, coronary calcification and findings of coronary CTA and PET predicted events equally (*P* = 0.477).

**Figure 4:**
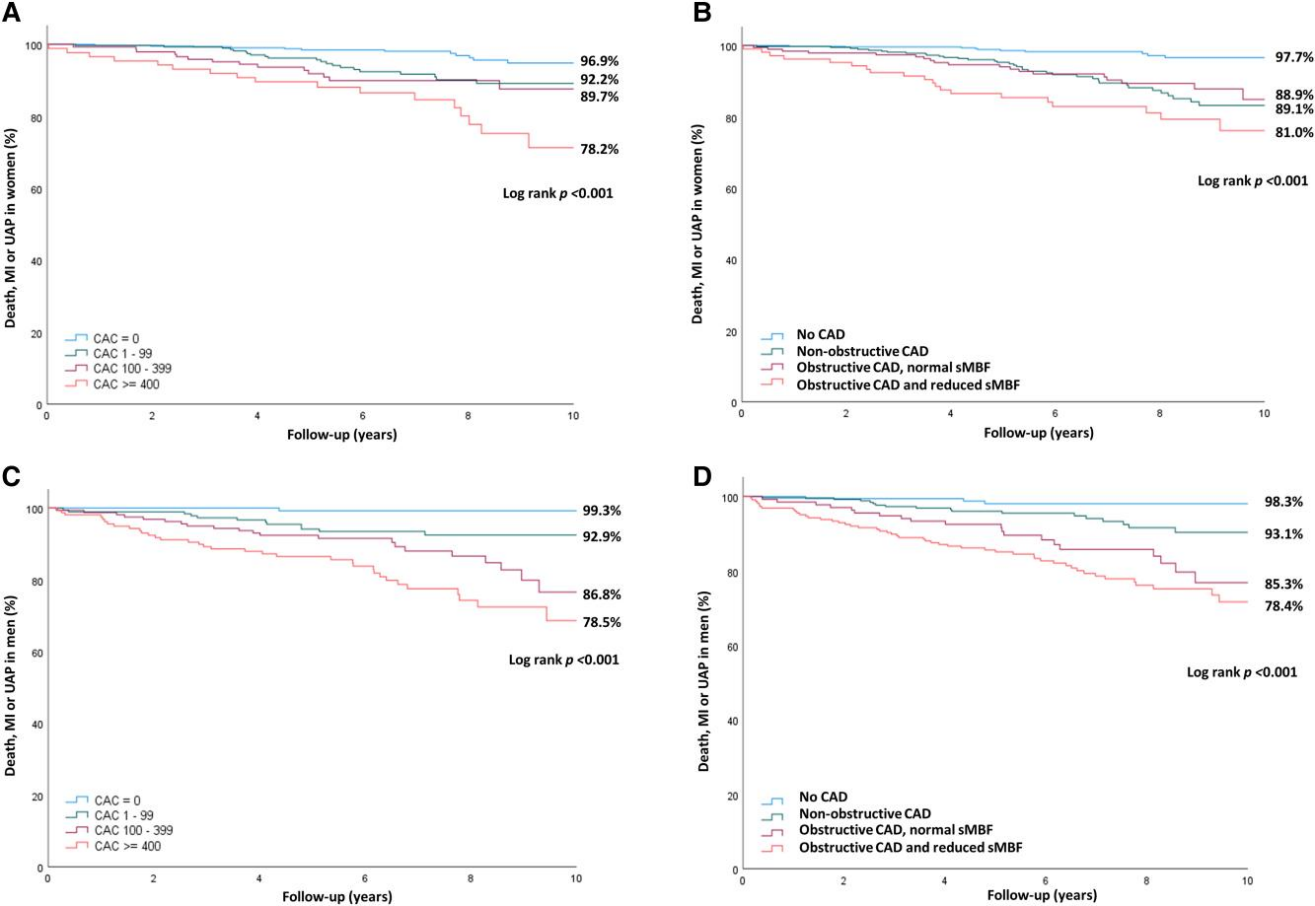
Kaplan–Meier curves comparing the occurrence of death, non-fatal MI or UAP according to coronary artery calcium (CAC) score (*A* and *C*) or combined coronary CTA and ^15^O-water PET (*B* and *D*) in women (*A* and *B*) and men (*C* and *D*).

*Figure 4* shows Kaplan–Meier estimates of the cumulative incidence of the composite events that were significantly different (*P* > 0.001) according to the extent of CAC and findings of PET-CTA in both men and women. Overall, the event rate was higher in men than women (Log-rank *P* = 0.02) with the annual adverse event rate being 1.37% in the whole cohort, 1.65% in men, and 1.17% in women.

## Discussion

This study compared the findings and prognostic value of combined coronary CTA and PET myocardial perfusion imaging between women and men referred to diagnostic testing for suspected obstructive CAD. The main finding of our study is that coronary CTA combined with ^15^O-water PET myocardial perfusion imaging predicts adverse events equally well in both women and men.

Our results are in line with recent studies showing that among patients evaluated for suspected CAD, men have more coronary atherosclerosis, a higher prevalence of obstructive CAD, and lower event-free survival than women.^4,5,6^ Unlike some recent analyses,^4,16^ our results do not show differences in referral for ICA or early invasive management between women and men with myocardial ischaemia. The observed revascularization rate is in line with previous studies.^11^ Although we do not have data on medical management and adherence to preventive therapies, our results suggest that the higher event rate in men is due to more extensive CAD.

Risk stratification is an important goal of diagnostic testing for CAD. Previous analyses have pointed out that there is limited data on differences in the prognostic value of noninvasive diagnostic tests for CAD, particularly coronary CTA, according to sex.^1^ More recently, retrospective studies have reported equal predictive value of per vessel extent of CAD by coronary CTA in women and men.^17^ In the Scottish Computed Tomography of the Heart (SCOT-HEART) trial, the addition of coronary CTA to a standard evaluation of patients with chest pain showed similar benefits in women and men.^5^ In the Prospective Multicenter Imaging Study for Evaluation of Chest Pain (PROMISE) trial, women appeared to derive even more prognostic information from CTA than men who tend to derive similar prognostic value from both anatomical and functional tests.^18^ Our results are in line with these previous studies demonstrating the prognostic value of a diagnostic strategy based on initial coronary CTA in both women and men.

The finding that CAC and non-obstructive CAD were predictive of events is consistent with the prognostic significance of atherosclerotic plaque.^19^ In a subgroup of patients in whom obstructive CAD was excluded by coronary CTA alone, the presence of non-obstructive CAD was associated with an increased event rate only in women. However, the number of patients was relatively small and the degree of atherosclerosis was small (77% of patients having CAC score <100) in this subgroup.

Combined or hybrid imaging enables the evaluation of both coronary anatomy by CTA and the detection of myocardial ischaemia by perfusion imaging. Previous evidence supports the diagnostic and prognostic value of SPECT perfusion imaging in women and men.^1,3,20,21^ Studies have also reported the similar prognostic value of myocardial perfusion abnormalities detected by ^82^Rb or ^13^N-ammonia PET perfusion imaging in women and men.^20,21^ Instead, there is limited evidence on the prognostic value of hybrid imaging. Two studies have shown that in patients with intermediate coronary lesions, evidence of ischaemia is associated with an increased event risk after hybrid imaging.^10,22^ However, possible differences in the prognostic value of combined imaging between women and men have remained unexplored. Our study included a large cohort of patients evaluated for suspected obstructive CAD by coronary CTA followed by ^15^O-water PET. Instead of all patients, stress MBF was evaluated by ^15^O-water PET in 657 patients (45% women) who had suspected stenosis detected by coronary CTA. ^15^O-water PET showed abnormal sMBF more often in men than in women (63% vs. 36%). Abnormal sMBF was associated with an increased risk of death, MI, or UAP in both women and men (HR 5.0 and 5.6, respectively). Our study extends previous findings in that there was no interaction between sex and the prognostic value of imaging findings based on a hybrid imaging strategy using coronary CTA combined with selective PET perfusion imaging.

### Study limitations

Our study is associated with all limitations of retrospective analysis in a cohort where imaging findings were reported to treating physicians. Previous studies suggest differences in the intensity of secondary prevention in men and women,^4,16^ but in this retrospective study, it was not feasible to evaluate changes in medication after imaging. Furthermore, since results of laboratory tests were available for a limited number of patients, some potentially relevant prognostic factors, such as anaemia, blood cholesterol or glucose levels, or renal dysfunction could not be included in the analyses. We defined abnormal perfusion as any segment with sMBF below the ischaemic threshold based on previous studies showing the predictive value of this parameter.^12,23^ The severity and extent of perfusion abnormalities may add prognostic information, but thresholds have not been established for ^15^O-water PET^23,24^ and our patient cohort was small for subgroup analyses. Reduced coronary flow reserve (CFR) has been associated with an excess risk of cardiovascular events in the absence of obstructive CAD, particularly in women.^25,26^ In our patient cohort, PET perfusion imaging was performed only for evaluation of haemodynamic significance of suspected obstructive lesions on coronary CTA and instead of CFR, only sMBF was assessed.^12^ Therefore, microvascular dysfunction in the absence of epicardial CAD as well as reduced CFR despite preserved sMBF remains undetected. However, we have previously published that the frequency of pure microvascular dysfunction is low in a similar cohort as in this study.^27^ Our study provides novel prognostic information about hybrid or combined imaging in women and men evaluated for suspected CAD.

## Conclusion

Combined anatomical and functional imaging shows more often normal coronary anatomy and less frequently ischaemic CAD in women than in men. Women have a lower rate of adverse events including death, MI, or UAP, after diagnostic testing. Findings of coronary CTA combined with PET myocardial perfusion imaging independently predict adverse events equally in women and men.

## Supplementary Material

jead072_Supplementary_Data

## Data Availability

The data underlying this article will be shared on reasonable request to the corresponding author.
