# Supplementary material for: Prognostic value of combined coronary CT angiography and myocardial perfusion imaging in women and men
Source: Eur Heart J Cardiovasc Imaging. 2023 Apr 22;24(9):1201–9. doi: 10.1093/ehjci/jead072 (PMC10445260; doi:10.1093/ehjci/jead072)
Supplement: jead072_Supplementary_Data [file jead072_supplementary_data.docx]

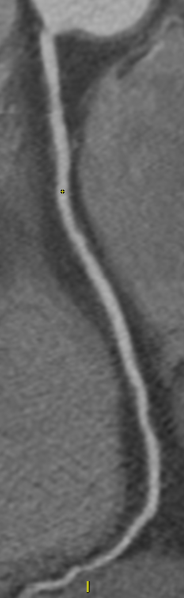

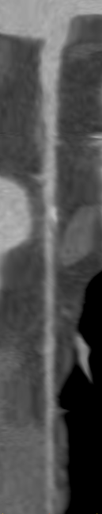

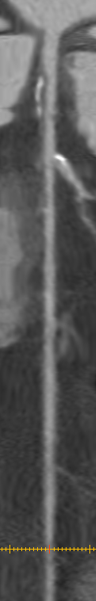

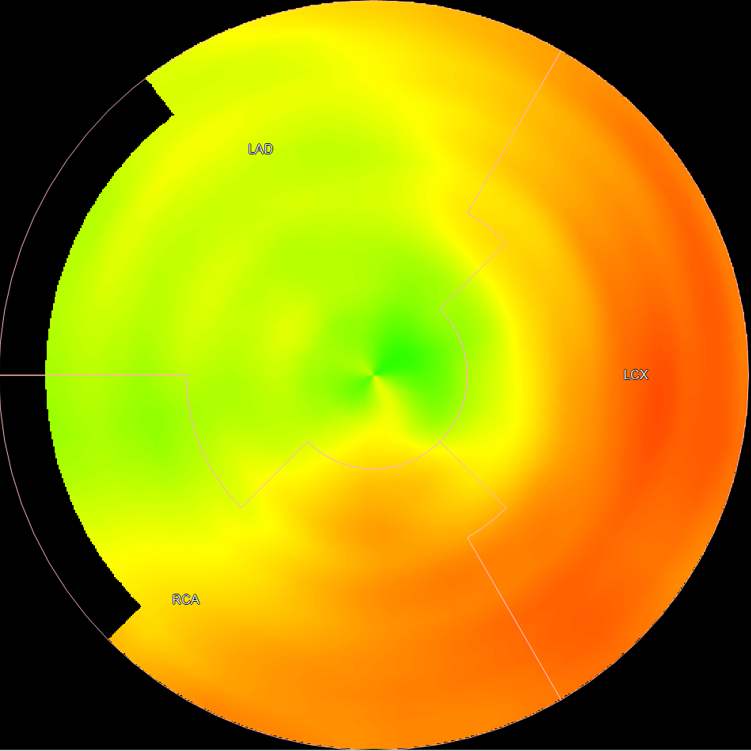

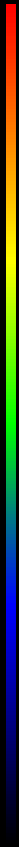


3.5

ml/g/min

0

ml/g/min

RCA

LCX

LAD

Supplemental image 1. 61 years old male who underwent CTA due suspected obstructive coronary artery disease. He had previous history of hypertension, smoking, high blood cholesterol and had a family history of premature CAD. He suffered from atypical angina. In CTA and PET MPI with ^15^O-water we can see that he had a intermediate stenosis in proximal LAD and related hypoperfusion. LAD sMBF was 1.9-2.1 ml/g/min, LCx 2.7 ml/g/min and RCA 2.6 ml/g/min. CAC 28. After imaging secondary preventative medications were prescribed and he had survived event free.


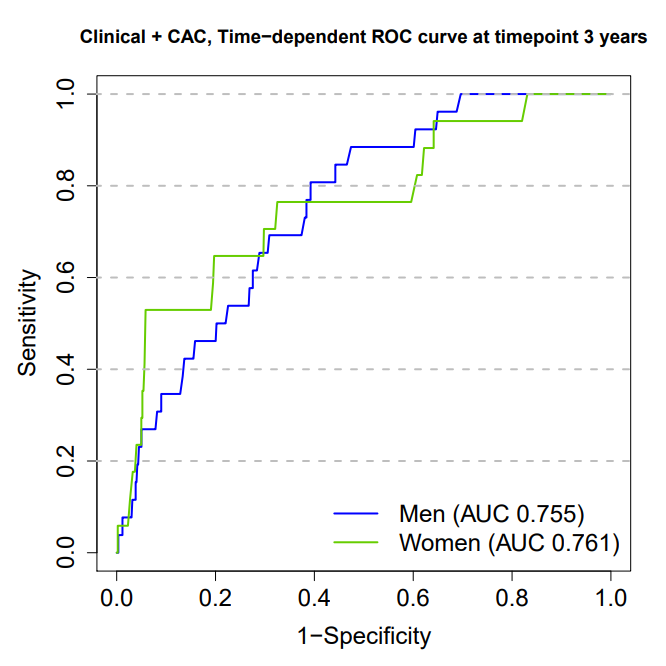

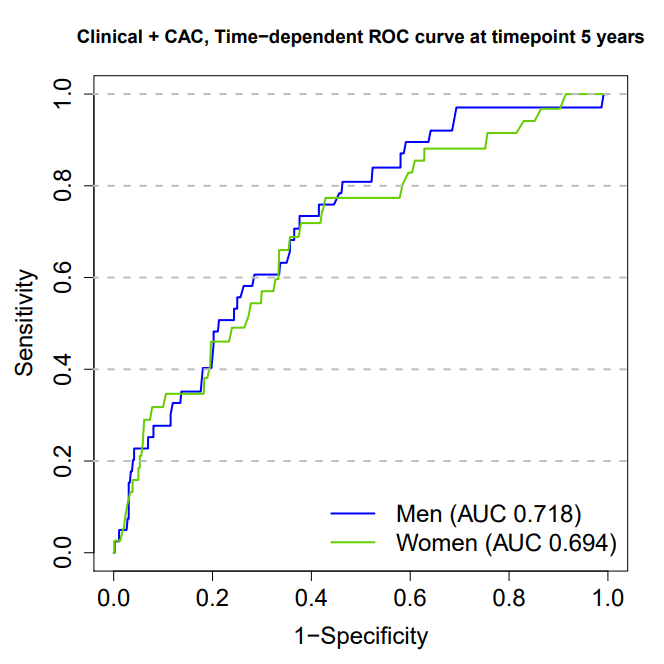

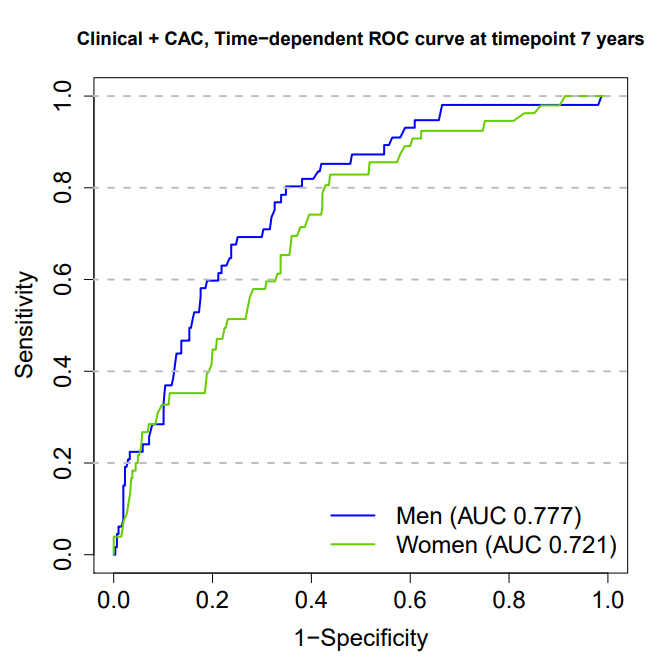

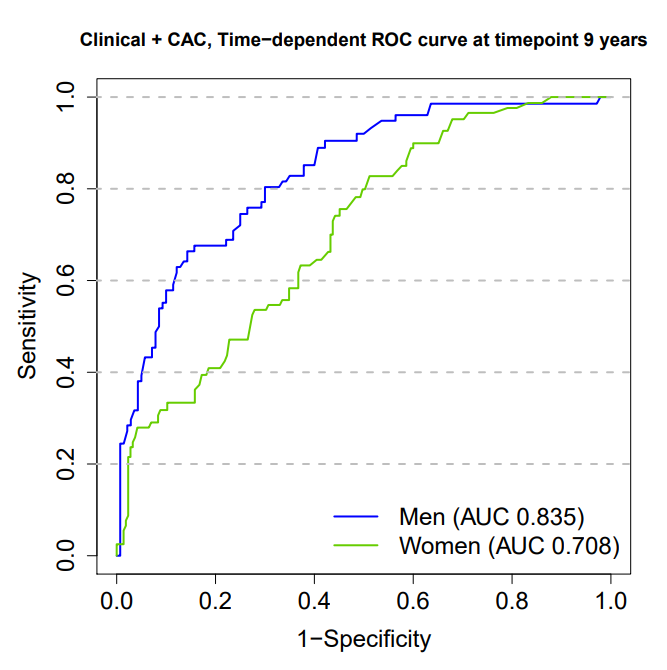

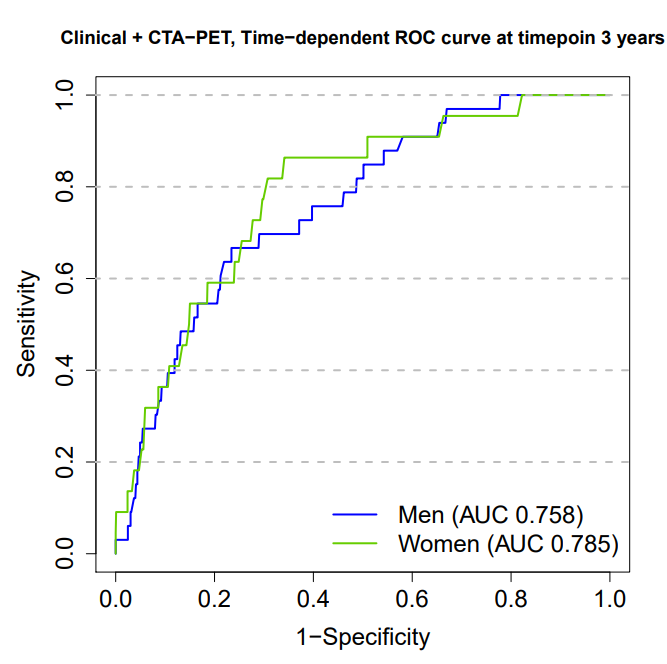

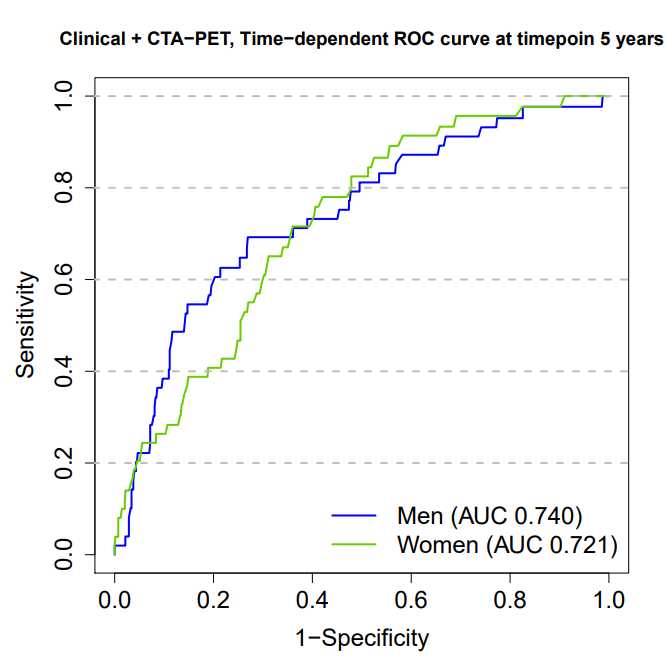

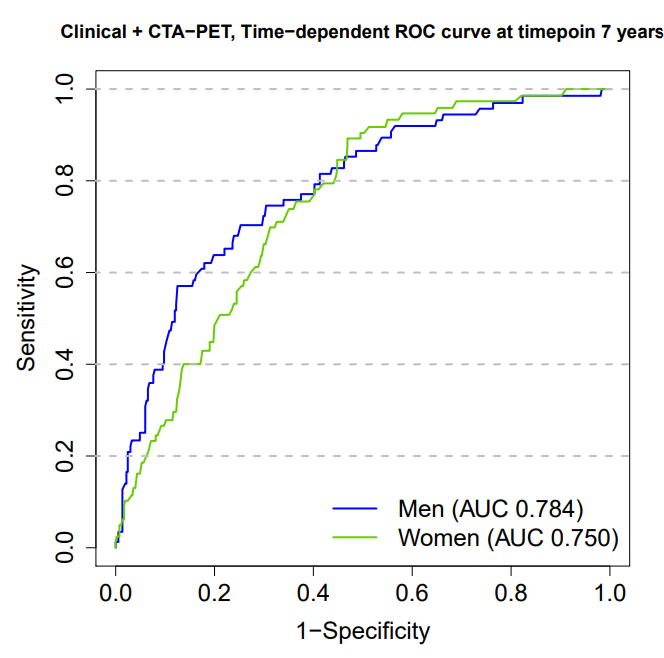

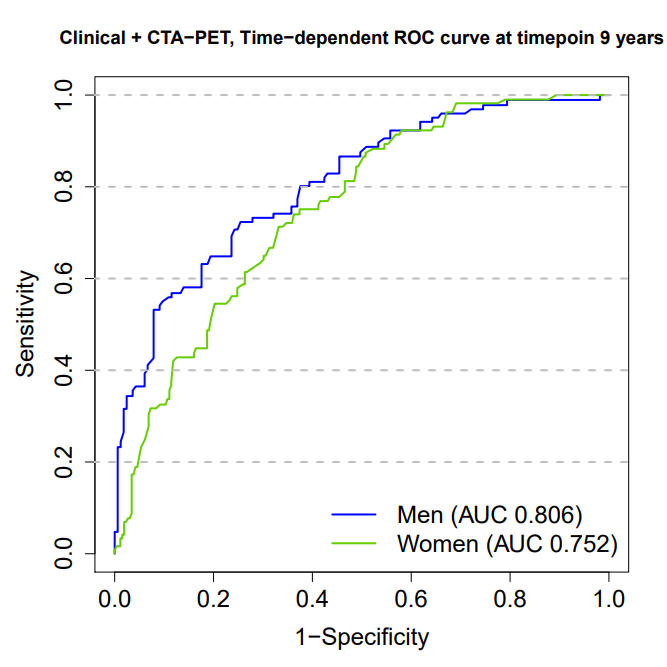


Supplemental figure 1. Time-dependent receiver operating characteristic (ROC) curves showing no difference in the performance of coronary artery calcium (CAC) score or combined coronary CTA and ^15^O-water PET in predicting composite end-point of death, non-fatal MI or UAP between men and women in different time points of 3,5,7 and 9 years. Analyses were adjusted with age, hypertension, and symptom status (typical angina and dyspnea).
